# Self-reported injuries and correlates among school-going adolescents in three countries in Western sub-Saharan Africa

**DOI:** 10.1186/s12889-022-13315-5

**Published:** 2022-05-05

**Authors:** Kwaku Oppong Asante, Henry K. Onyeaka, Nuworza Kugbey, Emmanuel Nii-Boye Quarshie

**Affiliations:** 1 Department of Psychology, School of Social Sciences, University of Ghana, University of Ghana, P. O. Box LG 84, Legon, Accra, Ghana; 2 Department of Psychology, University of the Free State, Bloemfontein, South Africa; 3 Department of Psychiatry, Massachusetts General Hospital/Mclean, Boston, USA; 4 University of Environment and Sustainable Development, Somanya, Eastern Region Ghana; 5 School of Psychology, University of Leeds, Leeds, UK

**Keywords:** Adolescents, Injuries, Trauma, School health, Unintentional injuries, West Africa

## Abstract

**Background:**

Unintentional injuries among adolescents constitute a significant public health problem globally. Injured adolescents may face negative outcomes ranging from poor academic performance to short- and long-term physical and psychosocial health struggles, and even death. The aim of this study was to estimate the prevalence and describe the correlates and most frequent causes of injuries among school-going adolescents in three West African countries – Benin, Ghana, and Liberia.

**Methods:**

We analysed self-reported data provided by 8,912 school-going adolescents who participated in the Global School-based Student Health Survey in Ghana (2012), Benin (2016), and Liberia (2017). Students responded to questions on sociodemographic factors, family involvement factors, mental health factors, school environment factors and injury behaviours.

**Results:**

The overall 12-month prevalence estimate of serious injuries in adolescents was 40.9% (Benin = 27.3%; Ghana = 46.1%; Liberia = 49.2%). The most frequently reported injury type was a broken bone or dislocated joint (33% in Benin), cuts or stab wounds (31.7% in Ghana), and non-specified injuries (35.2% in Liberia). Prevalence of serious injuries was higher among males and increased with age. In the multivariable logistic regression analysis, interpersonal aggression outside the family context (bullying victimisation, engaging in physical fights, and having been physically attacked) emerged as key correlates of increased odds of serious injuries.

**Conclusion:**

The relatively higher prevalence estimates of serious injury reported in this study underscore the need for the included countries to develop interventions aimed at reducing and preventing physical injuries among adolescents.

**Supplementary Information:**

The online version contains supplementary material available at 10.1186/s12889-022-13315-5.

## Background

Injuries during childhood and adolescence constitute an important global public health problem. They represent a leading cause of morbidity and mortality among adolescents and youth, particularly, in developing countries [1, 2]. Unintentional injuries have been shown to result in poor physical and psychological consequences for children and their families [3]. School-going adolescents who sustain injuries are likely to absent themselves from school resulting in adverse educational outcomes [4]. In addition to deaths, the literature also reports that a relatively large proportion of children who survive their injuries remain with temporary or permanent disabilities [5]. It is now well documented that the costs of hospitalisation, rehabilitation, loss of schooling, and loss of income to parents result from absence from work in order to care for an injured child [6, 7]. These associated adverse adolescent health and socio-economic outcomes require evidence-based intervention and prevention strategies that are targeted at specific risk and protective factors, particularly, in low resource settings, including countries in sub-Saharan Africa.

Evidence from previous studies in some countries in sub-Saharan Africa suggests that the 12-month prevalence estimate of serious injuries among school-going adolescents ranges from 38.6% in Swaziland [8], 55.7% in Mozambique [9], to 71.5% in Zambia [8]. In a recent study, the prevalence of serious injuries among 95,811 students who participated in the Global School-based Student Health Surveys (GSHS) in 47 low-income and middle-income countries was found to be about 40% during the previous 12 months [10]. Another cross-national study involving six African countries reported a relatively higher 12-month prevalence estimate of 68% among school going adolescents [8].

Some studies have reported significant associations between sociodemographic factors such as male gender [11, 12], and low socioeconomic status [2] with injuries. It has also been reported that several psychological and behavioural risk factors have been linked with injuries among adolescents. For example, adolescents who experience sleep difficulties and depressive problems are more likely to report injuries, compared to those without these problems [2, 12, 13]. Evidence also suggests that other factors such as substance use (including cigarette smoking and alcohol use) as well as factors within the school environment, such as bullying victimisation, truancy, physical fighting, and engagement in physical activity have been significantly associated with increased odds of sustaining serious injuries among adolescents [2, 8, 14, 15].

However, there is a paucity of research regarding the prevalence estimates and key psychosocial correlates of serious injuries among school-going adolescents in Western sub-Saharan Africa. So far in Africa, most studies conducted have focused on Eastern and Southern sub-Saharan African countries [8, 9]. Our systematic search of the literature did not show any published studies from countries in Western Africa on the prevalence of injuries and psychosocial correlates among nationally representative non-clinical samples of school-going adolescents.

Even so, clinical evidence (analysis of post-mortem records) from Ghana, for instance, revealed that 30% of adolescent injury related mortality were due to electrocution, poisoning, burns, stab/gunshot, hanging and other miscellaneous causes such as blast injury, traumatic injury from falling debris, and fall from height [16, 17]. In Liberia, alcohol consumption is associated with increased risk of several chronic medical conditions, including unintentional injuries and psychiatric co-morbidities in secondary school students [18]. Notably, however, no published data exists about the prevalence of serious injuries and correlates among in-school adolescents in Liberia.

Given the high burden of disability and related financial losses associated with injuries among young people [19], it is relevant that urgent measures are instituted to improve injury prevention. The identification of the specific behaviours and risk factors associated with injuries associated with school-going adolescents in various populations is an essential foundational step to the development and implementation of effective injury prevention education programmes [20, 21]. Accordingly, the goals of this study were to estimate the proportion of adolescents in three Western African countries (Benin, Ghana, and Liberia) who sustained serious injuries in the past 12 months, and to identify key factors associated with unintentional injuries in this population**.** Ultimately, it is hoped that the findings of this study will inform programmes and efforts at the creation of safe environments for young people (at least in the participating countries), thereby contributing broadly to the attainment of Goal 3 (good health and wellbeing) of the United Nations Sustainable Development Goals.

## Conceptual framework of adolescent injury

Adolescent (un)intentional injuries are multifactorial; injuries in young people could be due to the complex interplay of multilayered factors – individual, relationship, socio-cultural, and environmental factors [22]. Introduced in the 1970s [23], the ecological systems model has been applied to understand various public health issues among young people. Recently, the National Center for Injury Prevention and Control of the Centres for Disease Control and Prevention (CDC) has developed and applied the socio-ecological model as a framework for the understanding and prevention of injuries and violence among young people [24]. The socio-ecological model is derived from the broader ecological systems model. This study applies the socio-ecological model to understand school-going adolescents’ injuries. The socio-ecological model suggests that injuries in young people could be complex and result from a combination of multiple factors at the individual-level, relationship-level (including family and peers), community-level (including school), and societal-level [24]. The model underscores how an individual relates to others and the broader environment within which they live as a critical determinant of health outcomes. This means that in order to understand and develop evidence-informed prevention strategies and programmes related to (un)intentional injuries among adolescents, we need to consider the different layers of influence not only at an individual-level, but also at the immediate and the broader community and societal levels. The socio-ecological model has proven to be a useful theoretical framework for addressing several youth development and health outcomes, including injury. Among school-going adolescents, several factors may be associated with the susceptibility to injury. With the aid of a socio-ecological model, risk factors associated with adolescent injury can be grouped into four factors: 1) sociodemographic factors (male gender, older age and low socioeconomic status); 2) personal factors (psychological distress, tobacco use, alcohol use, truancy, and low peer support); 3) parental factors (parental supervision or low parental or guardian support); and 4) stressors from the school environment (bullying victimisation, physical fighting, and engagement in physical activity) [11]. On the basis of this framework, we expect school environmental factors (i.e. physical fighting, bullying victimisation, and engagement in physical activity), and personal factors (truancy, and substance use) to heighten the risk of serious injuries, whilst parental supportive behaviours may ameliorate the occurrence of serious injuries among school-going adolescents.

## Methods

### Design and context of study

The Global School-based Student Health Survey (GSHS) is a cross-sectional survey conducted in interested WHO member states to assess the behavioral risks and protective factors in multiple areas among school-going adolescent. For this study, the secondary data from the WHO and CDC Global School-based Student Health Survey (GSHS), from three West African countries were analysed: Benin, Ghana, and Liberia. These countries were chosen because their latest GSHS data (Benin [2016], Ghana [2012], and Liberia [2017]) were publicly available, when we planned this analysis. Also, these countries were chosen because from 2012 onwards, there were modifications to the questionnaires used within the Africa region, and data from these three countries were finalised and made publicly available. The study procedures were carried out in accordance with the Declaration of Helsinki and the research protocol was approved by the Ministries of Educations from Benin, Ghana and Liberia and the WHO.

Benin is a Francophone country of 11.5 million people, with 32.6% of the population aged less than 25 years [25, 26], with a low human development index (HDI rank of 163), mean years of schooling of 3.8 years, and life expectancy in 2018 of 61.5 years in 2018 [27]. It is considered a low-income country [28].

Ghana is an Anglophone country estimated to be inhabited by 30.9 million people, with 31.6% of the population aged 10–24 years [26, 29]. It is categorised as having a medium human development index (HDI rank of 176), with a life expectancy of 61.5 years in 2018, and mean years of schooling of 4.7 years [27]. Ghana is a lower-middle income country [28]. Liberia has a population of about 4.8 million people, with the population structure described as young: 63% is less than 25 years old and 32.8% is 10–24 years old [25, 26]. Life expectancy in the country is 63.7 years, with mean years of schooling of 4.7 years [27].

Liberia is an Anglophone country categorised as a low-income country, with a low human development index [HDI rank of 176] [27, 28]. Between 1989 and 2003, Liberia experienced a civil war which resulted in not only the destruction of infrastructure, including schools and healthcare, but also the torture, displacement and deaths of many citizens [30]. Recent evidence suggests that young people in post-conflict Liberia are increased risk of multifaceted social and health challenges, including risky sexual behaviours and substance use [31, 32].

### Sampling

The sampling strategy used in the participating countries for the GSHS has been reported elsewhere [33]. Briefly, a two-stage approach is used to generate a nationally representative sample of school children in the grades that educate the most students. In the first stage of the cluster sampling design, schools were randomly sampled from a list of all schools in the country using a probability proportionate to size method. This method ensured that the participants represented the geographic diversity of the country. In the second stage of the sampling process in each country, several classrooms that include high proportions of students from the targeted age groups were sampled for inclusion from within each of the participating schools. This allowed every student to have an equal chance of being selected for the study. Numerical weights were applied to each student record to enable generalisation of results to the eligible population. The sample included in this study were school-going adolescents in the three West African countries. Thus, out-of-school and non-school-going adolescents were not included in the study.

### Participation rates

As reported in a previous paper [33], 100% of the sample schools in Benin agreed to participate, and 78% of the students were sampled from Junior and Senior High School in the French school system. A total of 2,536 students participated in the Benin contribution to the GSHS. In Ghana, 100% and 96% of junior and senior secondary schools respectively agreed and participated in the study. Out of these schools, 82% of junior high school students and 74% of senior high students responded to the survey. A total of 3,632 students participated in the Ghana contribution to the GSHS. Finally, in Liberia, 98% of the sampled schools participated in the study and the student response rate was 73%, and the overall response rate was 71%. A total of 2,744 students participated in the Liberia GSHS.

### Measures

Informed by deductions from previous studies, socio-demographic variables, injury, school environment variables, parental involvement variables, mental health-related variables and personal factors were operationally extracted from the GSHS data of the participating countries. A summary of the independent variables derivation from survey data are presented in the supplementary Table.

#### Injury variables

The GSHS questionnaire includes three closed-ended questions about serious injuries. The first question asks about injury frequency (“During the past 12 months, how many times were you seriously injured?”). The next question asks about the type of injury (“During the past 12 months, what was the most serious injury that happened to you?”) and the final question asks about the primary cause of the injury (“During the past 12 months, what was the major cause of the most serious injury that happened to you?”). The response rates for serious injuries with each question yielded inconsistent results suggesting possible misclassification or a misunderstanding of the question by the students. The classification of each student as injured or not injured in the past year was determined with a two-step process. First, we calculated injury proportions separately for all three questions. Next, we then used Chi-square testing to evaluate differences in the proportions of injured students between the responses to the three sets of questions. In all the three countries, significant differences in proportion of injured school-going adolescents were observed when comparing the “frequency” question to the “type” or cause” questions. For example, the “frequency” question resulted in considerably higher positive responses to injury among adolescents than the “cause” or “type” questions in all three countries. However, we found no difference in proportion of seriously injured students when comparing the “type” and the “cause” questions. Therefore, to maintain consistency, students with positive responses to the question (“During the past 12 months, what was the most serious injury that happened to you?”) were classified as having serious injuries. This variable was recoded into a dichotomous variable and included in the analysis as an exposure variable. Given that the question about injury frequency asked about ranges of values (like “2 or 3 times”), it was impossible to estimate the specific counts as well as incidence rates of serious injury events over the past year.

#### Socio-demographics

Gender and age of the participants as captured in the survey, were the main demographic measures used. Age was categorised into two groups: 11–17 years and those aged 18 years and above.

#### School environment factors

In this study, five school-related factors were assessed. These include truancy – “During the past 30 days, how many days did you miss classes or school without permission?” Response options were ‘No’ (0 days) and ‘Yes’ (1–10 days); physical attack – “During the past 12 months, how many times were you physically attacked?” Response options were ‘No’ (0 times’ and ‘Yes’ (1–12 times); physical fight- “During the past 12 months, how many times were you in a physical fight?” Response options were ‘No’ (0 times) and ‘Yes’ (1–12 times); bullying—“During the past 30 days, how many days were you bullied?” Response options were ‘No’ (never — sometimes) and ‘Yes’ (most of the time, always); peer support – “During the past 30 days, how often were most of the students in your class kind and helpful?” Response options were ‘No’ (never — sometimes) and ‘Yes’ (most of the time; always) [2, 11, 33].

#### Family involvement factors

Parental and family involvement was assessed with five indicators: parental monitoring – “During the past 30 days, how often did your parents or guardians check to see if your homework was done?”; Parental understanding – “During the past 30 days, how often did your parents or guardians understand your problems and worries?”; parental bonding – “During the past 30 days, how often did your parents or guardians really know what you were doing you’re your free time?”; and parental intrusion of privacy – “During the past 30 days, how often did your parents or guardians go through your things without your approval?”; and hunger – “During the past 30 days, how often did you go hungry because there was not enough food in your home?” Response options were ‘No’ (never — sometimes) and ‘Yes’ (most of the time, always). These variables were scored with two response options—‘No’ (never — sometimes)’ and ‘Yes’ (most of the time, always). The conceptualisation of this question is guided by the definition of parental involvement especially among low socio-economic status and minority populations [11, 34].

#### Mental health

Mental health was assessed with two indicators: Feeling lonely — “During the past 12 months how often have you felt lonely?”. Response options were ‘No’ (never — sometimes) and ‘Yes’ (most of the time, always); Feeling worried (anxiety) “During the past 12 months how often have you been so worried about something that you could not sleep at night?”. Response options were ‘No’ (never — sometimes) and ‘Yes’ (most of the time, always) [35]

#### Personal and lifestyle factors

This variable was assessed with seven indicators: physical activity – “During the past 7 days, on how many days were you physically active for a total of at least 60 min per day?” Response options were ‘No’ (0 days) and ‘Yes’ (1-7 days); sexual behaviour – “During your life, with how many people have you ever had sexual intercourse”. Response options were ‘No’ (never-sometimes) and ‘Yes’ (1–6 or more); methamphetamine use – “During your life, how many times have you used amphetamine or methamphetamine (also called ice or yellow)?”. Response options were ‘No’ (0 times’ and ‘Yes’ (1–20 times); cannabis use – “During the past 30 days, how many times have you used marijuana?” Response options were ‘No’ (0 times) and ‘Yes’ (1–20 times); alcohol use – “During the past 30 days, on how many days did you have at least one drink containing alcohol?” Response options were ‘No’ (0 days) and ‘Yes’ (1–29 days; all 30 days); cigarette smoking – “During the past 30 days, how many days did you smoke cigarette?” Response options were ‘No’ (0 days’) and ‘Yes’ (1–29 days; all 30 days); and having a close friend – “How many close friends do you have?” Response options were ‘No’ (No friend) and ‘Yes’ (1–2; 3 or more) [36].

### Data analysis

Data for each country were analysed separately. Sample weights were applied in all analyses to reduce bias from non-response and improve generalisability to the population. To produce a sample that is equal to the original sample size and representative of the student population of each country, the numerical weight was adjusted by dividing each weight by the sum of weights and then multiplied by the sample size. All variables were re-coded on a dichotomous scale as in other existing GSHS studies [8, 9]. Descriptive statistics was used to provide prevalence and demographic estimates. Next, multivariable logistic regression analyses stratified by country were conducted to examine the independent predictors of serious injury. The results from the regression analyses are presented as adjusted odds ratios (OR) with 95% confidence intervals (CI). Statistical significance was defined as two-tailed *p*-value < 0.05 in all analyses. All analyses were conducted using STATA 15.0 statistical software. Participants with missing data for the “injury” variables were excluded from the final analysis.

## Results

### Socio-demographic characteristics

Data were collected from 8,912 students across the three GSHS-participating countries from the West African region (Table 1). The distribution of participants by age and sex was similar in all three countries. Students aged 18 years and above comprised 37.2% of the sample in Benin, 32.7% in Ghana and 50.2% in Liberia. The reported 12 month-prevalence estimates of serious injuries were 27.3% in Benin, 46.1% in Ghana, and 49.2% in Liberia. The overall total 12-month prevalence estimate of serious injury across all the three countries was 40.9%.

**Table 1:** Demographics of the participants in the Global School-based Student Health Survey (GSHS) in Benin, Ghana and Liberia, and the proportion of students who reported at least one serious injury in the previous 12 months

**Country**		**Benin (2016)**	**Ghana (2012)**	**Liberia (2017)**
Total number of GSHS participants		2536	3632	2744
Total number coded for injury status		2288	3227	2375
% of participants with a known injury status		90.2	88.8	86.6
% of participants with serious injury		27.3	46.1	49.2
GSHS participant % by Sex and Age	**Gender**	%	%	%
	Male	54.3	53.8	52.4
	Female	45.7	46.2	47.6
	**Age (years)**			
	≤ 11 years	0.3	1.1	2.4
	12 years	1.2	4.7	2.0
	13 years	5.0	7.7	3.8
	14 years	8.9	10.9	6.6
	15 years	13.2	13.8	8.0
	16 years	18.0	12.2	11.3
	17 years	16.2	16.9	15.7
	18 or more years	37.2	32.7	50.2
% Reporting Serious Injury by Sex and Age	**Gender**	***		
	Male	61.0	53.6	54.3
	Female	39.0	46.4	45.7
	**Age (years)**	*	*	
	≤ 11 years	0.3	0.8	2.8
	12 years	1.4	4.8	1.6
	13 years	6.1	7.4	4.0
	14 years	8.0	12.4	6.1
	15 years	13.3	14.0	8.0
	16 years	18.9	12.7	11.1
	17 years	18.1	17.2	16.3
	18 or more years	33.8	30.7	50.2

*Note*: *p < 0.05, p*** < 0.001

### Types and cause of injuries

The leading types and cause of injuries across all three countries are shown in Table 2. The most frequently reported injury type was a broken bone or dislocated joint (33%) in Benin, cuts or stab wounds (31.7%) in Ghana and other/no specified injuries (35.2%) in Liberia. Poisoning or taking too much of a drug was the least injury type in all the three countries accounting for 0.6% of reported injuries in Benin, 2.2% in Ghana and 2.6% in Liberia. Also, injuries from falls were the most frequently reported causes of injuries in Benin (47.8%) and Ghana (28.4%) whereas others/non-specified accounted for the most direct cause of serious injuries in Liberia (24.6%). The least reported direct cause of serious injuries in all the three countries was toxic inhalation or ingestion which constituted 0.5%, 3.5% and 2.9% in Benin, Ghana and Liberia respectively. Sex distribution of past 12-month serious injuries by countries is also shown in Table 2.

**Table 2:** Type and cause of most serious injury in the past year, by sex and country, among GSHS participants who reported at least one serious injury within the previous 12 months

	**Benin**		**Ghana**		**Liberia**	
	Female (%)	Male (%)	Female (%)	Male (%)	Female (%)	Male (%)
**Injury type**						
Broken bone or dislocated joint	29.1	35.5	20.4	26.0	12.3	19.8
Concussion or other head or neck injury, was knocked out, or could not breathe	7.9	10.9	12.1	7.0	10.3	9.2
Cut or Stab wound	12.0	7.4	27.0	35.5	23.0	32.3
Bad burn	0.0	1.1	2.6	1.7	3.7	1.2
Poisoned or took too much of a drug	12.5	3.7	6.6	4.9	7.2	4.0
Gunshot wound	6.5	15.4	1.9	3.4	3.5	2.0
Other/type not specified	32.0	30.0	29.3	21.5	40	31.5
**Cause of injury**						
I fell	52.1	45.2	29.5	27.6	16.1	17.5
Something fell on me or hit me	4.6	6.8	20.4	20.1	23.7	17.3
I was in a motor vehicle accident or hit by a motor vehicle	11.1	23.1	9.6	19.4	13.0	23.1
I was attacked or abused or was fighting with someone	5.8	1.8	5.7	6.1	13.2	10.7
I was in a fire or too near a flame or something hot	5.4	0.5	7.2	3.6	6.4	3.2
I inhaled or swallowed something bad for me	0.4	0.5	4.8	2.4	3.6	2.4
Other/cause not specified	20.6	22.1	22.8	20.9	24.1	25.7

## Correlates of serious injuries

The predictors of serious injuries are presented in Table 3. After controlling for other covariates, the only protective factor for serious injuries among adolescents in Benin was older age (OR = 0.66; 95% CI = 0.50 – 0.88; *p* = 0.004). Risk factors associated with serious injuries in Benin included having been attacked physically (OR = 2.02; 95% CI = 1.48 – 2.75; *p* < 0.001), having been bullied (OR = 2.00; 95% CI = 1.38 – 2.91; *p* < 0.001), anxiety (OR = 1.52; 95% CI = 1.11 – 2.07; *p* = 0.008), and alcohol use (OR = 1.46; 95% CI = 1.13– 1.89; *p* = 0.004).

**Table 3:** Predictors of Serious Injuries among school-going adolescents in three West African countries

**Variables**	**Benin**		**Ghana**		**Liberia**	
	Adjusted OR, 95% CI	*p*-value	Adjusted OR, 95% CI	*p*-value	Adjusted OR, 95% CI	*p*-value
**Demographics**						
Age (in years)						
11-17 years (Reference)	1.00		1.00		1.00	
18 years and above	**0.66 (0.50, 0.88)**	**0.004**	0.88 (0.72, 1.08)	0.227	1.08 (0.82, 1.42)	0.583
Gender						
Female (Reference)	1.00		1.00		1.00	
Male	1.24 (0.95, 1.61)	0.110	0.93 (0.77, 1.12)	0.459	1.01 (0.79, 1.30)	0.921
**School/environmental factors**						
Truancy	1.26 (0.93, 1.71)	0.131	1.14 (0.93, 1.40)	0.216	1.05 (0.81, 1.37)	0.709
Physical attack	**2.02 (1.48, 2.75)**	**< 0.001**	**2.04 (1.68, 2.49)**	**< 0.001**	**1.53 (1.17, 1.99)**	**0.002**
Physical fight	1.05 (0.77, 1.43)	0.771	**2.13 (1.73, 2.62)**	**< 0.001**	**1.67 (1.28, 2.19)**	**< 0.001**
Bullying	**2.00 (1.38, 2.91)**	**< 0.001**	**1.68 (1.23, 2.30)**	**0.001**	**1.90 (1.22, 2.94)**	**0.004**
Peer support	0.75 (0.55, 1.01)	0.061	0.96 (0.78, 1.18)	0.701	1.12 (0.86, 1.46)	0.398
**Family factors**						
Parental monitoring	1.21 (0.91, 1.60)	0.188	1.08 (0.87, 1.34)	0.464	1.17 (0.88, 1.56)	0.271
Parent-adolescent bonding	0.90 (0.67, 1.21)	0.484	0.94 (0.76, 1.17)	0.578	0.83 (0.62, 1.11)	0.208
Parental understanding	0.92 (0.68, 1.24)	0.574	0.96 (0.78, 1.18)	0.688	0.96 (0.72, 1.28)	0.802
Parental intrusion of privacy	0.98 (0.59, 1.65)	0.948	0.86 (0.66, 1.13)	0.271	0.78 (0.56, 1.09)	0.143
Hunger	1.07 (0.77, 1.49)	0.691	1.25 (0.94, 1.67)	0.121	1.24 (0.83, 1.84)	0.296
**Psychological factors**						
Loneliness	1.10 (0.76, 1.60)	0.618	**1.66 (1.28, 2.16)**	**< 0.001**	1.43 (0.74, 2.19)	0.097
Anxiety	**1.52 (1.11, 2.07)**	**0.008**	1.32 (0.99, 1.76)	0.058	1.35 (0.96, 1.91)	0.087
**Personal and lifestyle factors**						
Physical activity	1.08 (0.73, 1.61)	0.701	1.21 (0.98, 1.49)	0.071	**1.54 (1.17, 2.02)**	**0.002**
Sexual behavior	1.23 (0.93, 1.64)	0.144	1.19 (0.94, 1.50)	0.152	0.76 (0.57, 1.01)	0.055
Methamphetamine use	1.09 (0.42, 2.78)	0.862	**1.83 (1.07, 3.13)**	**0.027**	0.41 (0.16, 1.03)	0.057
Cannabis use	2.20 (0.76, 6.36)	0.146	1.71 (0.88, 3.33)	0.111	**3.23 (1.32, 7.87)**	**0.010**
Alcohol use	**1.46 (1.13, 1.89)**	**0.004**	1.24 (0.89, 1.71)	0.198	**1.39 (1.01, 1.93)**	**0.048**
Cigarette smoking	0.98 (0.53, 1.81)	0.949	0.57 (0.31, 1.06)	0.074	1.13 (0.56, 2.27)	0.733
Having a close friend	1.17 (0.79, 1.71)	0.433	**1.42 (1.06, 1.89)**	**0.017**	1.30 (0.87, 1.93)	0.201
**Hosmer–Lemeshow GOF test (sig)**	9.24 (0.322)		7.69(0.464)		12.85 (0.117)	
**McFadden’s pseudo R**^**2**^	0.064		0.098		0.065	

*GOF* Goodness of fit

In Ghana, school or environmental factors such as physical fighting (OR = 2.13; 95% CI = 1.73 – 2.62; *p* < 0.001), having been physically attacked (OR = 2.04; 95% CI = 1.68 – 2.49; *p* < 0.001), personal and lifestyle factors such as methamphetamine use (OR = 1.83; 95% CI = 1.07 – 3.13; *p* = 0.027) and bullying victimisation (OR = 1.68; 95% CI = 1.23 – 2.30; *p* = 0.001), psychological factors such as loneliness (OR = 1.66; 95% CI = 1.28 – 2.16; *p* < 0.001, and having a close friend (OR = 1.42; 95% CI = 1.06 – 1.89; *p* = 0.017) were the significant predictors of reporting serious injuries in the past 12 months (Table 3).

The results further showed that for students in Liberia, significant risk factors for injuries include school or environmental factors such as cannabis use (OR = 3.23; 95% CI = 1.32–7.87; *p* = 0.01), bullying victimisation (OR = 1.90; 95% CI = 1.22 – 2.14; *p* = 0.004), physical fighting (OR = 1.67; 95% CI = 1.28 – 2.19; *p* < 0.001), personal/lifestyle factors such as having engaged in physical activity (OR = 1.54; 95% CI = 1.17–2.02; *p* = 0.002), physical attacks (OR = 1.53; 95% CI = 1.17 – 1.99; *p* = 0.002), and alcohol use (OR = 1.39; 95% CI = 1.01–1.93; *p* = 0.048).

## Discussion

### Key findings

This analysis has shown three important findings: 1) the overall total 12-prevalence estimate of serious injury across all the three countries was 40.9%, with higher estimates reported among males and older adolescents than females and younger adolescents; 2) the most frequently reported injury type was a broken bone or dislocated joint (33% in Benin), cuts or stab wounds (31.7% in Ghana), and non-specified injuries (35.2% in Liberia) and 3) our multivariable logistic regression analysis showed interpersonal aggression outside the family context (bullying victimisation, engaging in physical fights, and having been physically attacked) as key correlates of increased odds of serious injuries, even though some lifestyle and psychological factors also showed statistically significant associations with serious injuries.

### Injury prevalence

In this study we found that, overall, approximately 4 in 10 adolescents reported a serious injury during the previous 12 months. The relatively higher prevalence estimates reported in these countries make serious injuries among adolescents a major public health issue which needs the attention of expansive research and intervention and prevention efforts. The 12-month overall prevalence estimate of serious injury (40.9%) across the three countries studied is similar to estimates reported in earlier studies using data from some low and middle-income countries [8, 10]. For instance, a study among adolescents from 47 low- and middle-income countries found a 12-month prevalence estimate about 40% [10]. However, in the current study there are variations in the reported 12- month prevalence estimates of serious injuries from the three countries, with the lowest rate found in Benin (27.3%) and the highest rate found in Liberia (49.2%). These variations in the West African countries included in this analysis reflect what pertains in other sub-Saharan African countries, as different prevalence estimates have been reported in different countries. For example, earlier studies have reported prevalence of serious injury in the past 12 months among school-going adolescents to be ranging from 38.6% to 71.5% in some southern African countries [8, 9].

We found variations in the types of injuries across the three countries with broken bone or dislocated joint being the most frequently reported injury in Benin, cuts or stab wounds in Ghana and other/non-specified injuries being the most frequently reported injury in Liberia. Plausible factors that help to explicate these differences are not readily available, discernible or speculative, considering that the measures used in this study were contextually less elaborated. Clearly, further studies, using more robust designs and measures, are needed to explore country-specific school and community/neighbourhood environmental factors related to injuries in this young population. Evidence from such studies will be potentially useful in designing targeted, context-sensitive and relevant prevention programmes. Nonetheless, the evidence of these differences could also be pointing to a need for specific prevention measures that target the most frequently reported injuries in these countries. It was observed that the 12-month prevalence of serious injury varied by gender as male adolescents reported higher prevalence in all the three countries and also occurrence of serious injuries decreases with age. Previous research among adolescents have found gender disparities in the prevalence of serious injuries [11, 12]. These findings suggest that age-sensitive interventions need to be implemented to address the risk factors associated with injury among younger adolescents as they are likely to engage in risky behaviours and be victims of bullying leading to serious injuries than older adolescents.

### Main subtype of injury

Broken bone or dislocated joint, and cuts or stab wounds emerged in this analysis as the most frequently reported subtypes of serious injuries among in-school adolescents. Given that the nature of these subtypes of injuries are often linked to violence, the evidence could be indicative of three realities: firstly, that interpersonal violence and aggression is widespread among in-school adolescents, particularly, in low- and middle-income countries, including those in Western sub-Saharan Africa [37, 38]; secondly, that the evidence could be a reflection of the negative impact of the exposure of adolescents in these countries to – culturally sanctioned – violence and aggressive behaviours [22, 37, 39] and lastly, that the evidence could be pointing to the vulnerability of adolescents as road users – i.e., cyclists, pedestrians, motorcyclists – in low- and middle-income countries [38, 40]. Even so, these could be potential targets for intervention and prevention programmes; for example, investment in pedestrian safety measures and policies, and the legislation and implementation of road user laws.

### Correlates of serious injuries

Within the socio-ecological model, the findings of our secondary analysis suggest that some lifestyle and psychological factors have statistically significant associations with serious injuries, but interpersonal aggression outside the family context (i.e., bullying victimisation, engaging in physical fights, and having been physically attacked) emerged as key correlates of increased odds of serious injuries. Evidence among adolescents suggests physical bullying victimisation to be a significant predictor of serious injuries [14, 15]. Bullying in the form of physical attacks and fights could lead to falls, causing serious injuries [2, 8, 11]. Anti-bullying policies and their effective implementations are needed in the various schools in the three countries to reduce the risk of serious injuries among adolescents.

Similarly, still at the interpersonal level, the finding of being physically attacked, and involvement in physical fights as significant correlates of serious injuries among adolescents supports evidence from both high-income counties and low- and middle-income contexts [2, 12–14]. Plausibly, this finding underscores the need for the institution and enforcement of anti-violence policies in schools. Additionally, at the individual level, age-appropriate interpersonal conflict resolution skills training is likely to be effective towards reducing and preventing adolescent violent behaviours, including physical attacks and fighting [41]. Lastly, the evidence of alcohol use as a strong correlate of serious injuries is not entirely surprising, even though it is consistent with previous findings from both high-income and other low- and middle-income countries [2, 8, 42]. Alcohol use as a predictive factor for serious injuries among adolescents could be attributed mainly to the adolescents’ access to alcohol in everyday life within the three countries studied. Although these countries have laws and policies that prohibit the access and sale of alcohol to underage individuals, the implementation remains a critical challenge [43, 44]. Clearly, governments of these countries need to enforce fully the laws and policies of alcohol use, particularly, those barring the retailing of alcoholic beverages to children and adolescents.

#### Strengths and limitations of the study

A key strength of this study is that this is one of the first studies to have used a large dataset from West African countries to examine adolescents’ serious injuries and their associated factors; thus, advancing our knowledge and contributing to addressing a gap in the scientific literature. Notwithstanding these strengths, some limitations should be noted. The GSHS study provides a cross-sectional database, thus, we could not infer causality between the identified associated factors and serious injuries. Refining the response items for the injury questions to clarify the prevalence of “Other” answers may help improve the quality of the injury type and *cause* data gathered, as it is not clear the specific type of adolescent injury. Providing further explanation to this item will make the GSHS even more useful for preventive health professionals and others committed to improving the safety of children and adolescents. Another critical limitation related to the vague nature of some of the variables in this analysis is the failure of the survey to include items assessing intentionality to inflict harm/injury on self or another person. Recent evidence suggests, for example, that drug poisoning (as a subtype of unintentional self-injury) has a strong risk association with self-harm among adolescents [45], while some injurious outcomes among adolescents are likely to be intentional and self-inflicted [16, 17]. Although a complex aspect of violence behaviour and injurious acts, intentionality has been identified by the WHO and leading researchers as an important element in the study of injury and violence-related behaviours [38].

Additionally, there is evidence to suggest that young people are inclined to report false verdict of their unintentional injuries [46]. We suspect that some of our participants might have provided socially desirable responses to the survey questions related to their unintentional injuries, while the responses by others might have been prone to recall bias, as the time lag between the occurrence of the injury and the survey might have been long. This may have equally led to the risk of biased results.

Also, it is imperative to interpret and adopt the findings of this analysis with caution, considering that we included only national level school-based datasets; enrolment rates may differ across key factors, including age, gender, income level, urbanicity, and rurality, as well as across the included countries. Similarly, the study included only students who were present at school on the day of the survey; besides plausible variations in attendance rates, it is also possible that some student absentees on the day of the survey were suffering from (serious) injuries. Taken together, these factors might have presented as potential sources of bias to this analysis, including possible underestimation of the prevalence of self-reported injuries, and the importance of some correlates. It is important for future studies to consider these broader factors.

## Conclusion

This study investigated adolescent serious injury and its associated factors among school-going adolescents in three West African countries – Benin, Ghana, and Liberia. The analysis found that a relatively higher prevalence of adolescent injuries in the three West African countries. The findings of this study support existing evidence that injuries are a common health outcome of interpersonal violent behaviours among school-going adolescents worldwide and that there are many exposures and risk behaviours linked to injuries that can be effectively addressed through health education and other low-cost, effective intervention and prevention programmes. Since there are different risk factors that influence school-going adolescent injury in each of the three West African countries studied, development of appropriate intervention should take into consideration a more differentiated approach. 

## Supplementary Information

**Additional file 1.**

## Data Availability

The datasets used and/or analysed during the current study are freely available from the WHO website: https://www.who.int/ncds/surveillance/gshs.

## Abbreviations

CDC: Centres for Disease Prevention
GSHS: Global School-based Student Health Survey
HDI: Human Development Index
WHO: World Health Organization
