# Supplementary material for: Self-reported injuries and correlates among school-going adolescents in three countries in Western sub-Saharan Africa
Source: BMC Public Health. 2022 May 5;22:899. doi: 10.1186/s12889-022-13315-5 (PMC9074264; doi:10.1186/s12889-022-13315-5)
Supplement: Supplementary file 1 — Additional file 1. [file 12889_2022_13315_MOESM1_ESM.doc]

**Supplementary Table**

**Independent variables derivation from survey data**

| Variable | Survey question | Original response options | Recoded | Benin, N = 2536 | Ghana, N = 3632 | Liberia, N = 2744 |
| --- | --- | --- | --- | --- | --- | --- |
| Age | How old are you? | 11–17 years (coded categorically) | N/A | N= 2535 | N= 3613 | N= 2661 |
| Sex | What is your sex | 1 = male; 0 = female | N/A | N= 2517 | N= 3594 | N= 2635 |
| Anxiety | During the past 12 months, how often have you been so worried about something that you could not sleep at night? | 1 = never to 5 = always | 1–3 = 0 and 4–5 = 1 | N= 2524 | N= 3617 | N= 2638 |
| Loneliness | During the past 12 months, how often have you felt lonely? | 1 = never to 5 = always | 1–3 = 0 and 4–5 = 1 | N= 2530 | N= 3618 | N= 2634 |
| School truancy | During the past 30 days, how many days did you miss classes or school without permission? | 1 = 0 days to 5 = 10 or more days | 1 = 0 and 2–5 = 1 | N= 2525 | N= 3577 | N= 2460 |
| Bullied | During the past 30 days, how many days were you bullied? | 1 = 0 days to 7 = all 30 days | 1= 0 and 2–7 = 1 | N= 2338 | N= 3292 | N= 2353 |
| Physically attacked | During the past 12 months, how many times were you physically attack? | 1 = 0 times to 8 = 12 or more times | 1 = 0 and 2–8 = 1 | N= 2531 | N= 3595 | N= 2661 |
| In a physical fight | During the past 12 months, how many times were you in a physical fight? | 1 = 0 times to 8 = 12 or more times | 1 = 0 and 2–8 = 1 | N= 2529 | N= 3620 | N= 2672 |
| Sexual risk behavior | During your life, with how many people have you ever had sexual intercourse | 1 = never had intercourse to 7 = 6 or more people | 1 = 0 and 2–7 = 1 | N= 2460 | N= 3410 | N= 2258 |
| Close friends | How many close friends do you have? | 1 = 0 friends to 4 = 3 or more close friend | 1 = 0 and 2–4 = 1 | N= 2523 | N= 3587 | N= 2627 |
| Peer support | During the past 30 days, how often were most of the students in your class kind and helpful? | 1 = never to 5 = always | 1–3 = 0 and 4–5 = 1 | N= 2524 | N= 3574 | N= 2409 |
| Current smoking of cigarette | During the past 30 days, how many days did you smoke cigarette? | 1 = 0 days to 7 = all 30 days | 1 = 0 and 2–7 = 1 | N= 2517 | N= 3548 | N= 2549 |
| Amphetamine use | During your life, how many times have you used amphetamine or methamphetamine (also called ice or yellow) | 1 = 0 times to 5 = 20 or more times | 1 = 0 and 2–5 = 1 | N= 2035 | N= 3339 | N= 2350 |
| Alcohol use | During the past 30 days, on how many days did you have at least one drink containing alcohol?” | 1 = 0 times to 7 = all 30 days | 1 = 0 and 2–7 = 1 | N= 2271 | N= 3358 | N= 2484 |
| Cannabis use | During the past 30 days, how many times have you used marijuana (also called weed, Jah, Indian hemp, ahabammmono, and ganja)?” | 1 = 0 days to 5 = all 30 days | 1 = 0 and 2–5 = 1 | N= 2503, | N= 3498 | N= 2383 |
| Parental Monitoring | During the past 30 days, how often did your parents or guardians check to see if your homework was done? | 1 = never to 5 = always | 1–3 = 0 and 4–5 = 1 | N= 2523 | N= 3593 | N= 2453 |
| Parental Intrusion of privacy | During the past 30 days, how often did your parents or guardians go through your things without your approval? | 1 = never to 5 = always | 1–3 = 0 and 4–5 = 1 | N= 2496 | N= 3585 | N= 2428 |
| Parent Understanding | During the past 30 days, how often did your parents or guardians understand your problems and worries? | 1 = never to 5 = always | 1–3 = 0 and 4–5 = 1 | N= 2525 | N= 3566 | N= 2402 |
| Parental bonding | During the past 30 days, how often did your parents or guardians really know what you were doing you’re your free time? | 1 = never to 5 = always | 1–3 = 0 and 4–5 = 1 | N= 2522 | N= 3499 | N= 2351 |
| Hunger | During the past 30 days, how often did you go hungry because there was not enough food in your home? | 1 = never to 5 = always | 1–3 = 0 and 4–5 = 1 | N= 2525 | N= 3621 | N= 2641 |
